# SENSORY PROCESSING CORRELATES WITH DEPRESSION AND PERCEIVED STRESS IN OLDER ADULTS

**DOI:** 10.1093/geroni/igac059.2534

**Published:** 2022-12-20

**Authors:** Megan Chang, Chiung-ju Liu, Erna Blanche

**Affiliations:** San Jose State University, San Jose, California, United States; University of Florida, Gainesville, Florida, United States; University of Southern California, Los Angeles, California, United States

## Abstract

Sensory processing is defined as the ability to respond to sensory information from the environment and to act accordingly to the situational demands. Sensory processing is associated with anxiety in middle-aged adults, specifically in those with sensory over-responsiveness and under-responsiveness. It remains unclear how age-related change in sensory processing is correlated with mental health. The purpose of this study is to examine the correlations between sensory processing patterns, depression, and perceived-stress in older adults. Respondents were recruited from community networks serving older adults. They were asked to complete an electronic survey, including the Adult Sensory Processing Scale (ASPS), the Perceived Stress Scale, and the Center of Epidemiologic Depression Scale-Revised. ASPS has 11 factors related to over-responsive, under-responsive, and sensory seeking in visual, auditory, tactile, vestibular and proprioceptive input.Of 148 older adults (Mean age = 72 years) completed the survey, 30% perceived moderate to high levels of stress, and 18% had depressive symptoms. The total score of the ASPS Scale is positively correlated with perceived stress (r=0.26; p=.001) and depression (r=0.27; p=.001). Specifically, those who were over-responsive to auditory and vestibular input, and under-responsive to proprioception had higher stress levels and greater depression.Sensory decline or impairment in older adults may alter older adults’ ability to process sensory information. As sensory processing has significant impact on anxiety and perceived stress in older adults, it should be considered in evaluation and intervention, particularly on audition, vestibular and proprioception. Including sensory-based approach may help better manage their mental health.

